# Extra gastrointestinal stromal tumor (EGIST) of the ischioanal space detected on pelvic MRI

**DOI:** 10.1016/j.radcr.2026.06.045

**Published:** 2026-06-30

**Authors:** Manizhe Ataee Kachuee, Iman Mohseni, Mahshid Panahi, Peyman Family, Ali Saadat Rashti, Neda Azarpey, Yasaman Sharifi

**Affiliations:** a Department of Radiology, School of Medicine, Firoozgar Hospital, Iran University of Medical Sciences, Tehran, Iran; b Firoozgar Clinical Research Development Center (FCRDC), Iran University of Medical Sciences, Tehran, Iran; c Department of Pathology, Firoozgar Hospital, Iran University of Medical Sciences, Tehran, Iran; d Department of Radiology, School of Medicine, Iran University of Medical Sciences, Tehran, Iran

**Keywords:** Gastrointestinal stromal tumors (GIST), Extragastrointestinal stromal tumor (E-GIST), Ischioanal region, C-Kit negative

## Abstract

Gastrointestinal stromal tumors (GIST) are sub epithelial soft tissue neoplasms in the gastrointestinal tract. Extragastrointestinal stromal tumors (E-GISTs) are rare, comprising 5%-7% of GISTs. A case of a 65-year-old female with abdominal bloating, heaviness, and constipation is reported. Magnetic resonance imaging revealed a mass in the ischioanal region, initially thought to be a solitary fibrous tumor. The mass was completely excised, and pathological examination confirmed it as a C-Kit Negative E-GIST. This case highlights the rarity and uniqueness of the ischioanal mass detected through pelvic MRI.

## Introduction

Gastrointestinal stromal tumors (GIST) represent a distinct subtype of subepithelial soft tissue neoplasms within the gastrointestinal tract (GIT). They are the most prevalent mesenchymal tumors of the gastrointestinal tract, predominantly arising in the stomach and small intestine [1]. Extragastrointestinal stromal tumors (E-GISTs) are exceedingly rare neoplasms, constituting about 5%-7% of all gastrointestinal stromal tumors (GISTs) [2]. EGISTs are predominantly located in the omentum, mesentery, retroperitoneum, pelvic region, abdominal wall, liver, gallbladder, pancreas, bladder, seminal vesicle, vagina, prostate [3], and scrotum [4]. Two decades ago, the diagnosis was often leiomyoma, schwannoma, or leiomyosarcoma; however, advancements in molecular techniques and immunohistochemistry have rendered the diagnosis of GIST more accurate and comparatively straightforward [5]. Immunohistochemically testing reveals continuous expression of CD117 (c-kit protein) and CD34 [6,7]. The suggested imaging modalities include computed tomography (CT), magnetic resonance imaging (MRI), and positron emission tomography (PET). The conventional management of limited, resectable, and nonmetastatic GISTs and EGISTs involves surgical intervention, supplemented by imatinib in cases of high risk, recurrence, or metastasis [8,9]. C-kit-negative gastrointestinal stromal tumors (GISTs) are rare, and there is limited data about the diagnosis and treatment of c-kit-negative GISTs [10]. Here, we have encountered a c-kit-negative EGIST case in the ischioanal fossa, initially classified as a solitary fibrous tumor, and we will report on its diagnosis and treatment.

## Case history/examination

A 65-year-old Iranian female arrived with stomach bloating, heaviness, and constipation as her early symptoms. Symptoms such as anorexia, nausea, vomiting, pain, or weight loss were notable absences. The patient exhibited no significant past medical, surgical, or familial history and was otherwise in good health. Furthermore, the individual denied any use of tobacco, alcohol, recreational drugs, or any relevant psychosocial history. The clinical examination revealed that the vital signs were within normal limits. Abdominal examination revealed no masses or tenderness. Blood tests, including a full blood count, urea and electrolytes, and liver function tests, were performed and yielded unremarkable results within normal limits.

## Differential diagnosis, investigations, and treatment

**Fig. 1:**
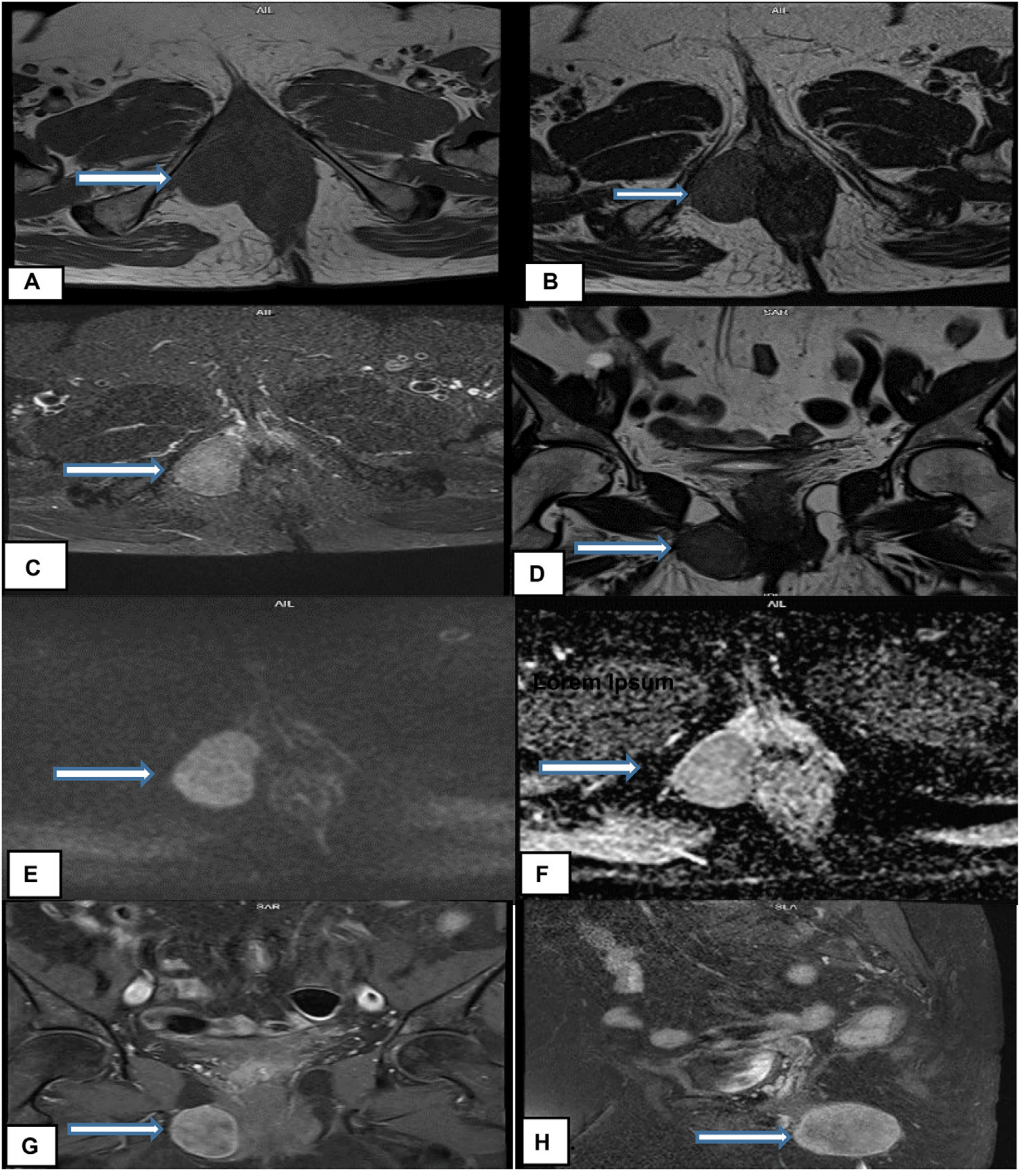
Magnetic resonance imaging of the ischioanal mass (white arrow). (A) Axial T1 weighted. (B) Axial T2 weighted. (C) Axial short tau inversion recovery (STIR). (D) Coronal T2 weighted. (E) Axial diffusion-weighted imaging (DWI). (F) Axial apparent diffusion coefficient (ADC) mapping. (G) and (H) postcontrast axial and sagittal imaging. A well-circumscribed iso to high T1W and intermediate T2W in the ischioanal space, which seemed to be intramuscular without invasion to adjacent structures such as the rectum.

The workup included an abdominal ultrasonography that reported normal abdominal organs. Other Imaging work-up of MRI with rectal-pelvic protocol revealed a well-circumscribed iso to high T1W and intermediate T2W in the ischioanal space, which seemed to be intramuscular without invasion to adjacent structures such as the rectum (Fig. 1). Additional Chest x-ray revealed normal. A possible diagnosis based on imaging studies was the first solitary fibrous tumor and neurogenic tumors or EGIST could be other possibilities.

## Conclusion and results (outcome and follow-up)

**Fig. 2:**
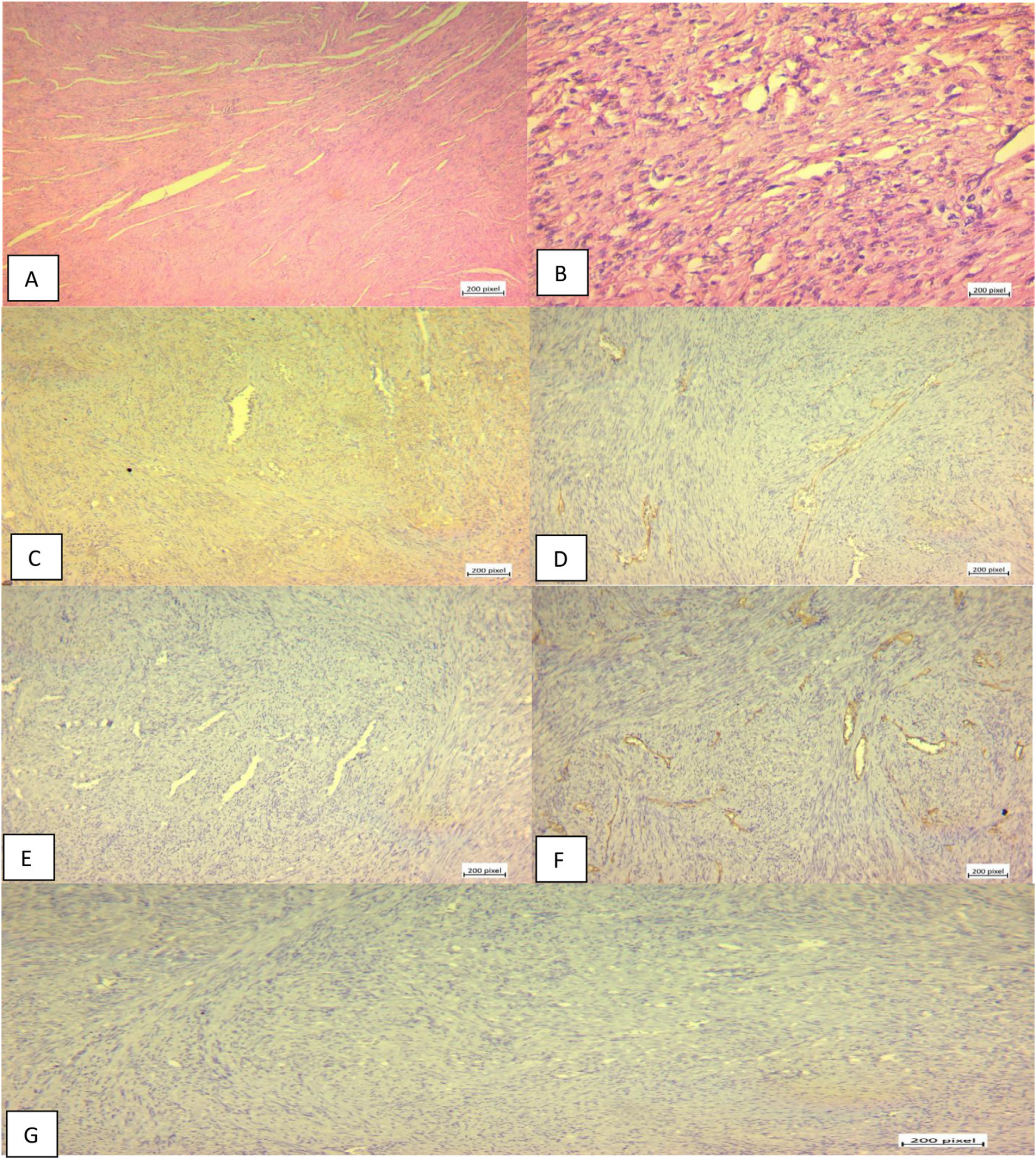
Pathologic assessment of C Kit negative EGIST. (A) Lesion composed of bland spindle cells with elongated nuclei and eosinophilic cytoplasm, arranged in fascicles. (medium power). (B) short fascicles of spindle cells with a moderate amount of eosinophilic cytoplasm without obvious nuclear atypia (high power). (C) DOG 1 IHC staining. (D) Desmin IHC staining. (E) C kit IHC staining. (F) CD34 IHC staining. (G) STAT6 IHC staining.

The tumor was resected, and the pathologic reports were a 5.5 × 5 × 4.5 cm tumoral mass with a smooth surface and creamy homogenous appearance. Sections revealed neoplastic tissue composed of spindle cells with a mitotic rate of less than 2/50 HPF. IHC staining showed negative reactivity for CD117, Desmin, CD34, S100, STAT6, and betacatenin and positive reactivity for Dog1 and Ki67. All are in favor of C Kit Negative EGIST (Fig. 2). Subsequent treatment with imatinib has been administered. The patient exhibited no recurrence in the following imaging assessments.

## Discussion

Gastrointestinal stromal tumors (GISTs), derived from gastrointestinal mesenchymal tissue, represent a minor fraction of abdominal soft tissue neoplasms. Conversely, EGISTs are classified as mesenchymal tumors that arise external to the gastrointestinal tract, comprising less than 5% of GIST cases [11,12]. The anatomical locations of EGIST that have been documented recently include the retroperitoneum, mesentery, and omentum. Limited investigations have documented EGIST arising from mullerian structures, however the exact prevalence of these areas has not been described till now [13]. The usual tumor diameter at diagnosis is 8 cm, although GISTs have been documented to reach sizes of up to 40 cm [14,15]. The reported median age is approximately 65 years [15]. The clinical presentation is highly diverse and mostly depends on the location and size, among the most important considerations. Symptoms may include nausea, vomiting, gastrointestinal bleeding, anemia, abdominal pain, and abdominal mass [15].

As demonstrated in this patient's case, conventional imaging techniques like CT or MRI frequently produce inconclusive findings when the EGIST is situated in atypical regions, such as the ischioanal space. One possible differential diagnosis based on our case imaging study was a solitary fibrous tumor of ischiorectal space that usually exhibits low signal intensity relative to muscle on T1-weighted images and variable signal intensity on T2-weighted images.

GISTs originate from interstitial cells of Cajal and exhibit 3 histological types: spindled, epithelioid, and mixed-type cells [16]. The detection of CD117 expression, commonly referred to as kit, using immunohistochemistry (IHC) stating is essential for the diagnosis of GISTs. CD117 expression is seen in more than 95% of GISTs [17]. Nonetheless, CD117 expression is absent in 5% of c-kit-negative GISTs. In these cases, supplementary staining discovered on GIST-1 (DOG1, also referred to as ANO1) may be beneficial for confirming a GIST. The diagnosis and treatment of c-kit-negative GISTs remain ambiguous [18]. Currently, surgery and imatinib should be employed as a treatment approach for c-kit-negative GISTs.

Herein, we present a case of a 65-year-old woman exhibiting abdominal distension and constipation, who underwent a rectal protocol MRI. The results revealed a well-circumscribed lesion, iso to high on T1-weighted imaging and intermediate on T2-weighted imaging, located in the ischioanal space, appearing to be intramuscular without invasion of adjacent structures, including the rectum, suggestive of a solitary fibrous tumor. The mass was completely resected, and pathological evaluations indicated a c-kit negative EGIST. The rarity of the site of the indicated case and the probable differential, which are identical in imaging studies with solitary fibrous tumor, as well as the need of pathologic confirmation and its significance for early chemotherapy treatment, are the instructional lessons of this case.

## Author contribution

MAK, YSH, IM, PF, NA, and ASR: wrote the manuscript; MP reviewed pathologic points and comments on expertise visions; MAK, IM, PF, and YSH reviewed and interpreted the MRI; MAK, YSH, IM, PF, and ASR reviewed and edited the manuscript. All authors read and approved the final manuscript.

## Patient consent

Written informed consent was obtained from the patient for publication of this case report and accompanying images. A copy of the written consent is available for review by the Editor-in-Chief of this journal on request.
